# The Safety of FeedKind Pet^®^ (*Methylococcus capsulatus*, Bath) as a Cultured Protein Source in the Diet of Adult Dogs and Its Effect on Feed Digestibility, Fecal Microbiome, and Health Status

**DOI:** 10.3390/ani15131975

**Published:** 2025-07-04

**Authors:** Matt Longshaw, Bradley Quest, Walt Miller, Patricia M. Oba, Olivia R. Swanson, Kelly S. Swanson, Kathryn Miller

**Affiliations:** 1 Calysta (UK), Ltd., Wilton Centre, Redcar TS10 4RF, UK; 2 BSM Partners, 83 W Champions Boulevard, Rogers, AR 72712, USA; drquest@bsmpartners.vet (B.Q.); drmiller@bsmpartners.vet (K.M.); 3 Charles River Laboratories, 1407 George Road, Ashland, OH 44805, USA; walt.miller@crl.com; 4 Department of Animal Sciences, University of Illinois Urbana-Champaign, Urbana, IL 61801, USA; obapm@illinois.edu (P.M.O.); ors2@uic.edu (O.R.S.); ksswanso@illinois.edu (K.S.S.)

**Keywords:** target animal safety, single cell protein, microbial protein, postbiotic, nutrition, diet formulation, microbiome, digestibility

## Abstract

Thirty-two healthy adult dogs (16 males and 16 females) were fed control kibble diets for one month, followed by six months (Weeks 0 to 25) of diets containing either 0, 4, 6, or 8% cultured protein derived from *Methylococcus capsulatus* (FeedKind Pet^®^, FK), then they were fed control diets (0% FK) for a further two months (Weeks 25 to 34). The diets were isonitrogenous, isolipidic, and isocaloric and stage- and age-specific. The dogs were assessed for overall health, weight gain, and body condition score (BCS). Blood samples were collected 1 week prior to randomization, during acclimation, then in Weeks 5, 13, 25, 30, 32, and 34 for hematology, coagulation, and clinical chemistry; urine was collected according to the same time schedule for urinalysis. Feces were assessed for parasite load and presence of occult blood during Weeks 5, 9, 13, 17, 21, and 25. Fecal samples were collected during acclimation and Weeks 25 and 34 for fecal microbiome analysis and in Week 25 for apparent total gastrointestinal tract digestibility (ATTD). All dogs maintained a healthy weight and BCS throughout the study. Hematology parameters were within normal limits at the end of each phase of the study. With the exception of a decrease in serum phosphorus level and in urine pH in all groups at the end of the study, urine and serum chemistry results were within normal limits at the end of each phase. ATTD values for organic matter, protein, and energy exceeded 80%, whilst digestibility values for copper were around 20%. The fecal microbiome was dominated by Firmicutes. Alpha diversity increased during the safety phase before returning to baseline levels during the washout phase. The dominant genera in all groups were *Megamonas*, *Peptoclostridium*, *Turicibacter*, *Catenibacterium*, *Fusobacterium*, *Romboutsia*, and *Blautia*. The study has shown that the inclusion of cultured protein at up to 8% of the total diet of adult dogs can provide sufficient nutrition and is safe with no long-term effects on a range of health parameters.

## Simple Summary

Thirty-two beagles were fed diets containing up to 8% cultured protein for six months, then fed control diets for a further two months. The safety of the protein was assessed by measuring blood and urine parameters as well as the growth of the dogs. Additionally, the intestinal microbiome was shown to be positively influenced by the cultured protein. The diets were well tolerated by the dogs with no reduction in feed intake and were readily digested, providing all of the essential nutrients required. The study showed that FeedKind Pet^®^ is safe as a protein source for dogs and can be included at up to 8% of the total diet with no harmful side effects.

## 1. Introduction

There is a steady increase in demand for high-quality protein sources of pet food from pet parents, creating competition with the human food system and challenging our current food structure’s sustainability [1]. It is estimated that by 2050, the population will have increased by 35%, causing the need for food to increase by 70% and meat production to increase by 100% from needs in 2010 [2,3]. This is further exacerbated by using protein resources to feed companion animals. In 2020, it was estimated that the mean annual global land use to produce dry pet food was 49 megahectares (Mha). This is approximately twice the land area of the United Kingdom [4]. To help alleviate these strains on the food system, there is an opportunity for the pet food industry to explore alternative protein sources that are both nutritionally appropriate and sustainable. Full nutrition profiles, including macro- and micronutrient listings, amino acid content, digestibility assays, target animal safety testing, and palatability studies are a few important considerations when evaluating alternative protein sources for pet food. This will help to ensure that a complete and balanced diet can be properly formulated. We hypothesize that cultured protein is a safe, sustainable, and environmentally friendly alternative protein source for complete and balanced dog food.

A well-established alternative to traditional meat-based proteins in pet food is plant protein sources [5]. Unfortunately, plant protein is not always a complete protein. For example, it does not supply any significant amount of the amino acid taurine, which is an essential nutrient for cats, so diets made with plant proteins must be fortified properly to make them complete and balanced for pets [6]. Plant proteins also can be lacking in other essential sulfur-containing amino acids such as cystine and methionine. Several studies have shown that commercial plant-based pet foods may not be complete and balanced, even though they claim to be, and require fortification to sustain their limitation in minerals and amino acids [7,8]. Other studies have shown that properly formulated plant-based protein diets are a viable alternative protein source for dogs [9,10]. Plant proteins may be more environmentally sustainable than traditional animal proteins [11]. However, a potential shortcoming is that they may not match the nitrogen metabolism of animal proteins; they may require complementary proteins to supplement to meet all amino acid requirements, and the variability in amino acid digestibility requires that they are carefully formulated to avoid deficiencies [12,13,14,15].

Insects appear to be a promising alternative as their life cycle is short, and they have a high turnover and biomass conversion rate [16]. In addition, they have been shown to be a highly palatable alternative to traditional protein sources in dog food [17,18]. Several studies have demonstrated that dogs not only accept insect protein well, but that insect protein can have high digestibility and good amino acid composition when compared to traditional animal proteins [17,19,20,21]. Yet the production of insect protein and its use in the pet food industry is still faced with some challenges, notably among which is customer perception. However, there are also ethical aspects like species-specific killing methods, mass-rearing conditions, and transportation [22]. The risks of foodborne microbial contamination, allergenic responses, palatability, and long-term safety for pets still need to be demonstrated [23,24].

Single-cell organisms such as bacteria, yeasts, fungi, and microalgae [25] are gaining more and more acceptance as protein sources, known as single-cell proteins (SCPs). Due to their high productivity and their approval for human consumption by the Food and Drug Administration (FDA) and the World Health Organization (WHO), SCPs have emerged as a promising alternative protein source for animal feed that scientists believe will play a vital role in altering the traditional food supply chain [26,27,28] by providing alternative sustainable sources [29]. Cultured protein, derived from *Methylococcus capsulatus*, has an amino acid profile suitable for fish, livestock, and companion animals, and can be used as a substitution for more conventional protein sources like fishmeal [30,31,32,33]. Cultured protein has been shown to be a safe alternative to fishmeal protein in numerous aquaculture species [34,35,36,37,38,39]. Additionally, it has demonstrated safety in feeding terrestrial species such as pigs [40,41,42,43,44,45], chickens [32,33,46,47,48], mink [32,49,50,51], and foxes [52,53]. Whilst these studies have shown that *M. capsulatus* is safe for use as a feed ingredient at the right inclusion level, other studies have shown that it can have detrimental effects, particularly when fed to excess. The effects noted include decreased feed intake due to reduced palatability, minor changes in overall weight, increased inflammatory responses, dysbiosis of the microbiome, urogenic effects due to higher levels of purines in the SCPs, and the increased size and weight of the mesenteric lymph nodes [38,43,54,55,56,57,58]. With the exception of purine effects and lymph node size, no other studies have demonstrated these effects in pets or in mammals. Importantly, although mesenteric lymph node weight and size increased at high inclusion levels of *M. capsulatus*, there was no concomitant histopathological damage noted in cats fed the cultured protein over a two-month period [58]. Although the underlying mechanism for these responses is not clear, it is believed that it may be driven by the presence of lipopolysaccharide in the cell wall or copper within the cell, both of which are immunomodulatory; lymph node enlargement is a normal response to antigens [58].

FeedKind^®^ Pet, FK (Calysta Inc., San Mateo, CA, USA), is composed of approximately 90% of an obligate methanotroph bacteria *Methylococcus capsulatus* Bath—alongside *Aneurinibacillus danicus*, *Brevibacillus agri*, and *Cupriavidus cauae*—which is grown on methane gas involving aerobic fermentation, and is processed using centrifugation, heat inactivation, and spray-drying into a highly concentrated protein source [59,60]. No growth media ingredients remain in the resulting biomass. These microorganisms, as a vegan protein source, offer several advantages to traditional proteins, with rapid growth and high protein content within a short time frame compared to traditional livestock farming. FeedKind has a high protein value (>65% protein, 5% fat, and 12% ash).

It is economical to use gas fermentation to produce protein because of its reasonable cost, low transportation cost, and sufficient supply [61]. Cultured protein production systems require significantly less land and water compared to conventional protein sources. Cultured protein can be a rich source of essential amino acids, vitamins, minerals, and other vital nutrients crucial for canine health with a balanced amino acid profile with high amino acid (AA) digestibility and DIAAS-like values > 100 [62]. In addition, cultured protein has numerous bio-active cofactors and offers a clear opportunity for value addition to the ingredient [54]. Some of these are well-known (e.g., n-3 LC-PUFA, nucleotides, or peptidoglycans), while others are less known [63]. Cultured protein potential as a pet food ingredient is promising based on results in mink and fox with regard to digestibility and palatability, but more studies are needed in dogs.

Despite these advantages, the use of cultured protein in pet food remains a relatively new concept and requires further research. The aim of this study was to evaluate the safety, bioavailability, digestibility, and microbiome effects of the FeedKind cultured protein as an alternative source of protein when administered to male and female adult dogs via 4%, 6%, and 8% dietary inclusions over a 6-month period with a 2-month period of recovery. The study design followed FDA guideline 185 [64] for a target animal safety study and included typical measures of variables such as physical examinations and observations, and clinical pathology tests including hematology, urinalysis, serum chemistry, growth, and health outcomes as well as microbiome analysis. Variations to the study design were agreed upon with the FDA prior to the commencement of the study. This included the addition of a two-month “recovery” period at the end of the safety study to determine if any changes that may have occurred in the study were reversible. We hypothesized that cultured protein is a safe, sustainable, and environmentally friendly alternative protein source for complete and balanced dog food. By integrating cultured protein into dog food formulations, we aim to create nutritionally complete and balanced diets that support pet health while addressing the pressing sustainability challenges associated with traditional protein sources.

## 2. Materials and Methods

### 2.1. Diets

The four test diets had the following FeedKind^®^ (FK) percentage inclusions and diet designations: 0% (FK0), 4% (FK4), 6% (FK6), or 8% (FK8). FK0 represents the control group diet whilst FK4-8 represents the test diets. FK mainly replaced the soybean meal component of the diet at 30, 37, and 53%, respectively, in the three test diets. The nutrient composition of FK is shown in Table 1 and the proximate composition and formulation of the test diets are shown in Table 2. Crude protein, crude fat, crude fiber, and ash concentrations were formulated to be the same among all diets and each diet met the Association of American Feed Control Officials (AAFCO) requirements for all life stages of the dog [65]. The extruded kibble diets were all produced on the same production line at Kansas City Treats (Kansas City, KS, USA). The diets were stored at 18–24 °C until the study commenced.

### 2.2. Animal Selection and Housing

Charles River Laboratories (Ashland, OH, USA) Animal Care and Use Committee approved all animal care procedures before animal experimentation. All methods adhered to the United States Public Health Service Policy on Humane Care and Use of Laboratory Animals. The dogs were immunized against a number of viral infections (parvovirus, distemper, adenovirus type 2, parainfluenza, and rabies) and bacterial infections (Leptospirosis, and *Bordetella*) prior to arrival at the testing facility. Thirty-six purebred beagles (5.8 to 10.4 kg) were acclimated for twenty-six days (Day −26 to Day −1). Four dogs (2 males and 2 females) were alternates for this study. Thirty-two intact adult dogs (16 males and 16 females) that were individually identified with an ear tattoo and a subcutaneously implanted electronic identification chip were included in the study (Day 1). Males and females were housed in separate rooms that were maintained at 19–24 °C with a relative humidity of 30–70% and a lighting regimen of 12L:12D. Mean age was 13–14 months on Day 1. Dogs were excluded from the study if they had reduced food consumption, were thin or had a low body weight, or if they had excessive changes in body weight that were ±10% of the initial body weight during the study. The provision of food other than that defined in the study also constituted grounds for exclusion.

### 2.3. Feeding Trial

Body weights (BWs) were measured in kilograms to 1 decimal point one week prior to the start of the study and then on a weekly basis (±2 days). The dogs were assigned a body condition score (BCS) on a scale ranging from 1 to 5 (where 1 was thin, 2 was lean, 3 was optimal, 4 was heavy, and 5 was very obese), which were conducted one week prior to randomization, on the day of randomization, on Day −1, then every 4 weeks until the end of the study. After excluding dogs with low food consumption or poor body condition, the remaining dogs were randomly assigned to one of the four dietary treatment groups (stratified by sex). Within each same-sex and treatment group, dogs were paired and randomly assigned to adjacent cages. The randomization was performed using SAS Release 9.4 (SAS Institute, Cary, NC, USA). Pair socialization occurred daily for 2–4 h within assigned pairs following the end of the daily feeding period.

Prior to acclimation (Day −26 to Day −1), the dogs were offered 300 g each of a standard lab diet then transitioned to a 50:50 mix of standard diet and control diet (FK0) for one week before being fed only the FK0 diet based on the desired kcal/day requirements (approximately 2.5–3.0% of body weight per day) for the remainder of the acclimation period. Starting on Day 1 (study start), 8 dogs (4 males and 4 females) continued on the control diet (FK0), and the remaining dogs were fed test diets containing either 4% (FK4), 6% (FK6), or 8% (FK8) cultured protein for 180 days (safety phase). Each of the study groups eating the test diets including FeedKind contained 4 males and 4 females. On Day 181 (recovery/washout period), the FK feeding was discontinued and all dogs were fed the FK0 diet until Day 239 when the dogs were transferred to the Charles River Ashland animal colony. Starting from Day −26, until the end of the study, weighed food was provided once daily at approximately 8:00 am. Any food remaining after at least three hours was removed, weighed, and recorded. To ensure that all the dogs were offered enough food to maintain their ideal BW, the desired kcal/day for each dog was calculated using the resting energy requirement for dogs [66], as per Equation (1) below:

70 × [(BW in kg)^0.75^ × 2] (1)

The diet amounts were adjusted weekly with the aim of maintaining an acceptable BCS and a body weight within 10% of each dog’s body weight at the beginning of the study. Feed conversion ratios (FCRs) were calculated using Equation (2), as below:

Food Consumed (kg)/[(Final Weight (kg) − Initial Weight (kg)](2)

FCRs were calculated for the whole study (Day 1 to Day 239), for the safety phase (Day 1 to Day 180), and for the recovery phase (Day 181 to 239). Weight gain (WG) (expressed as a %) was calculated using Equation (3), as below:

((Final Weight (kg) − Initial Weight (kg))/Initial Weight (kg)) × 100(3)

WG values were calculated for the same time periods as FCR. Fresh, clean water was available at all times. If necessary, due to clinical signs such as prolonged inappetence, a supplemental diet (e.g., canned food) or added water was provided to individual animals as recommended by the clinical veterinarian. The provision of supplemental feed was considered a reason to exclude that animal from the study. Study staff were blinded to dose group assignments throughout the study.

### 2.4. Clinical Examinations

Cage-side observations were performed by technicians at least twice daily (prior to feeding and again in the afternoon) throughout the study and were included but were not limited to general condition, general attitude, cognition, and the presence of emesis or abnormal urine or feces. Detailed clinical observations were performed by technicians weekly throughout the study and were included but were not limited to the evaluation of excreta, reaction to touch, muscle tone, respiration, coat, skin, eyes, ears, nose, mouth, genitals, anus, and any visible or palpable masses. Complete veterinary physical examinations by a licensed clinical veterinarian were performed one week prior to randomization, one week prior to Day 1 of the study, and during Weeks 5, 13, 25, 30, and 34 of the study. The examinations included evaluations of the ocular, musculoskeletal, cardiovascular, respiratory, nervous, integumentary, lymphatic, genitourinary, and gastrointestinal systems as well as the general behavior and gait of the animal. The evaluations (normal/abnormal) of the cardiovascular and respiratory systems included thoracic auscultation, heart rate, respiratory rate, mucous membrane color, and evaluation of capillary refill time. The examinations of the integumentary system included an otoscopic exam and evaluations of skin and hair coat. Lymphatic system evaluations included palpation of submandibular, prescapular, popliteal, and inguinal lymph nodes. Body temperature was also taken.

A complete ophthalmic examination was conducted on all dogs by a board-certified ophthalmologist prior to selection for the study and during Week 25. The ophthalmic exam included the use of an indirect ophthalmoscope and a slit lamp biomicroscope. Prior to each exam, each dog was treated with 1% tropicamide. Neurologic examinations were conducted on all dogs prior to selection for the study, and in Weeks 25, 30, and 34. These neurologic exams were conducted by a clinical veterinarian and included general attitude, behavior, and muscle function. Proprioception and postural reactions were evaluated via proprioceptive positioning, hemi-hopping, hemi-standing, wheelbarrowing, hooping, placing reactions (tactile), extensor thrust, placing reactions (visual), righting reactions, and eye tracking. Cranial nerves were evaluated via head movement, head symmetry, pupil symmetry, vestibular nystagmus, eye position, head muscle tone, palpebral reflex, pupillary light reflex, eye menace reflex, jaw tone, tongue test, and pharynx test. Spinal nerves were evaluated via muscle tone, flexor reflex, perineal reflex, panniculus reflex, and patellar reflex.

### 2.5. Sample Collection

Fasting blood samples were collected via venipuncture from the jugular vein. Two mL of blood was placed in a potassium EDTA blood tube for complete blood count (CBC) analyses; two mL of blood was placed in a sodium citrate blood tube to isolate plasma for coagulation analyses; and 1.5 mL of blood was placed in a serum separator blood tube for serum chemistry profile analyses. Blood samples were collected 1 week prior to randomization, in Week −1 during acclimation, then in Weeks 5, 13, 25, 30, 32, and 34.

In addition to general daily and weekly fecal assessments as described above, fecal samples from individual dogs were collected and assessed for the presence of parasites and/or their eggs, and for the presence of occult blood one week prior to randomization, during Week −1 of acclimation, and during Weeks 5, 9, 13, 17, 21, and 25. At Day −10 (during acclimation), Day 178, and Day 235, a target weight of 5(±2) g of fresh feces from each dog was collected and placed in a single collection unit per subject. The samples were stored in a freezer set to maintain −70 °C before being shipped in a single batch to the University of Illinois for fecal microbiome analysis. From Day 176 to Day 180, dogs in all four study groups were individually housed and all feces were collected and weighed. After weighing, feces from each dog were placed in sealed, labeled containers and stored at −20 °C until required for the determination of apparent total tract digestibility.

Urine from all dogs was collected overnight using urine collection pans at Week −1 during acclimation, then at Weeks 5, 13, 25, 30, 32, and 34.

### 2.6. Analytical Methods

#### 2.6.1. Proximates

Third-party laboratory testing (Midwest Laboratories, Omaha, NE, USA) was conducted on the finished test diets at the end of the study (20 months after production) (Table 3). As product shelf life was determined to be 18 months, this sampling point was considered to be beyond this date. Values were determined using AOAC methods [67] as follows: moisture (AOAC 930.15), amino acids (AOAC 994.12), cysteine and methionine (AOAC 994.12), tryptophan (AOAC 988.15), fatty acids (AOAC 996.06), crude protein (AOAC 990.03), acid hydrolysis fat (AOAC 954.02), crude fiber (AOAC Ba 6a-05), ash (AOAC 942.05), water soluble vitamins [68], and peroxide values determined using HPLC. Heavy metals (arsenic, cadmium, lead, and mercury) and aromatic hydrocarbons in the cultured protein source were determined using ICP-MS.

#### 2.6.2. Hematology and Coagulation

CBC analyses for each dog at each time point was performed using an Advia 2120i (Siemens Healthineers, Malvern, PA, USA) and included red blood cell count (10^6^/µL), hemoglobin concentration (g/dL), hematocrit (%), mean corpuscular volume (fL), red blood cell distribution width (%), mean corpuscular hemoglobin concentration (g/dL), mean corpuscular hemoglobin (pg), reticulocyte count (10^9^/L), platelet count (10^3^/µL), mean platelet volume (fL), white blood cell count (10^3^/µL), neutrophil count (10^3^/µL), lymphocyte count (10^3^/µL), monocyte count (10^3^/µL), eosinophil count (10^3^/µL), basophil count (10^3^/µL), and large unstained cells (10^3^/µL).

Blood coagulation parameters were analyzed using Stago STA Compact Max Coagulation (Stago, Parsippany, NJ, USA) and included activated partial thromboplastin time (seconds), fibrinogen (mg/dL), and prothrombin time (seconds). All data were compared with in-house (Charles River Laboratories (CRL)) reference ranges for males and females older than 12 months.

#### 2.6.3. Clinical Chemistry

Serum clinical chemistry profiles were analyzed by an Advia 1800 (Siemens Healthineers, USA) and included alanine aminotransferase (ALT) (U/L), aspartate aminotransferase (AST) (U/L), alkaline phosphatase (U/L), gamma-glutamyltransferase (U/L), creatine kinase (U/L), total bilirubin (mg/dL), blood urea nitrogen (mg/dL), creatinine (mg/dL), calcium (mg/dL), phosphorus (mg/dL), bicarbonate (mEq/L), bile acids (mg/dL), lactate dehydrogenase (U/L), total protein (g/dL), albumin (g/dL), globulin (g/dL), albumin/globulin ratio (A/G), glucose (mg/dL), cholesterol (mg/dL), triglycerides (mg/dL), sodium (mEq/L), potassium (mEq/L), and chloride (mEq/L). All data were compared with in-house (CRL) reference ranges for males and females older than 12 months.

#### 2.6.4. Urinalysis

Urinalysis parameters, obtained with the Advia 1800 (Siemens Healthineers, USA), included color, appearance/clarity, specific gravity (via refractometer), pH (via electrode measure), microscopy (including crystals, casts, red blood cells, white blood cells, and epithelial cells), volume, protein, glucose, bilirubin, ketones, and the presence or absence of blood. All data were compared with the test facility’s historical control reference ranges for males and females older than 12 months.

#### 2.6.5. Fecal Analysis and Digestibility Calculations

At the end of the study, the stored frozen fecal samples along with 250 g of each of the four test diets were sent to the University of Illinois for analysis. Fecal samples were dried at 55 °C in a forced-air oven for at least 72 h or until a constant weight was achieved to ensure the complete removal of moisture. Composite samples of the diet and dried fecal material from each animal were separately ground using a 2 mm screen in a Wiley Mill (Model 4, Thomas Scientific, Swedesboro, NJ, USA). Dry ice was incorporated at a ratio of 1:1 (by volume) during the grinding process to minimize nutrient degradation. The ground diet and fecal samples were analyzed for dry matter (DM) and ash content following the methods specified by the Association of Official Analytical Chemists [67] (DM: method 934.01; ash: method 942.05), with organic matter (OM) calculated accordingly.

Total lipid content was determined using acid hydrolysis and extraction methods facilitated by ANKOM Technology equipment (Hydrolysis System, XT15 Extractor, and RD Dryer; Macedon, NY, USA). Crude protein (CP) content was derived from total nitrogen values obtained via a Leco analyzer (TruMac N, Leco Corporation, St. Joseph, MI, USA; [67]. Gross energy was assessed using an oxygen bomb calorimeter (Model 6200, Parr Instruments, Moline, IL, USA). The total dietary fiber (TDF) content of the diets was measured using the method outlined by Prosky et al. [69]. Finally, copper content was analyzed according to AOAC [70] (method 968.08). Briefly, dry-ashed diet and fecal samples were solubilized in a 20% HCl solution and then quantified using flame atomic absorption spectroscopy (PerkinElmer, Shelton, CT, USA).

Data from the fecal and feed analysis in conjunction with the total amount of diet eaten and feces produced during the collection period by each dog were used to determine apparent total tract macronutrient digestibility (ATTD) values using Equation (4), as below:

[[nutrient intake (g per day) − fecal output (g per day)]/nutrient intake (g per day)] × 100(4)

Dietary nitrogen-free extract (NFE), expressed as a percentage, was calculated using Equation (5), as below:

100 − [Fat (%) + Crude Protein (%) + Ash (%) + Total Dietary Fiber (%)](5)

#### 2.6.6. Fecal Microbiota Analysis

Total DNA from feces was extracted using DNeasy Powersoil Pro Kit (Qiagen, Carlsbad, CA, USA). The concentration of extracted DNA was quantified using a Qubit 3.0 Fluorometer (Life Technologies, Grand Island, NY, USA). 16S rRNA gene amplicons were generated using a Fluidigm Access Array (Fluidigm Corporation, South San Francisco, CA, USA) in combination with the Roche High Fidelity Fast Start Kit (Roche, Indianapolis, IN, USA). The primers 515F (5′-GTGCCAGCMGCCGCGGTAA-3′) and 806R (5′-GGACTACHVGGGTWTCTAAT-3′) target a 252 bp fragment of the V4 region of the 16S rRNA gene and were used for amplification (primers synthesized by IDT Corp., Coralville, IA, USA) according to Caporaso et al. [71]. CS1 forward tag and CS2 reverse tag were added according to the Fluidigm protocol. The quality of the amplicons was assessed using a Fragment Analyzer (Advanced Analytics, Ames, IA, USA) to confirm amplicon regions and sizes. A DNA pool was generated by combining equimolar amounts of the amplicons from each sample. The pooled samples were size-selected on a 2% agarose E-gel (Life technologies, Grand Island, NY, USA) and extracted using a Qiagen gel purification kit (Qiagen, Valencia, CA, USA). Cleaned size-selected pooled products were run on an Agilent Bioanalyzer to confirm the appropriate profile and average size. Illumina sequencing was performed on a MiSeq using v3 reagents (Illumina Inc., San Diego, CA, USA) at the Roy J. Carver Biotechnology Center at the University of Illinois. Fluidigm tags were removed using the FASTX-Toolkit (version 0.0.14), and sequences were analyzed using QIIME 2, version 2023.7 [72] and DADA2 (version 1.14) [73]. The forward reads were truncated at 254 base pairs based on quality score profiles, and high-quality (quality value ≥ 20) sequence data derived from the sequencing process were demultiplexed. Chimeric sequences were identified and removed using the consensus method in DADA2. The DADA2 pipeline within QIIME 2 was used for denoising, dereplication, chimera removal, and the generation of amplicon sequence variants (ASVs) [73]. Taxonomy was assigned using the Naive Bayes classifiers trained on the Silva database (v.138) [74,75,76]. Singletons (ASV that are observed fewer than two times) and ASV that have <0.1% of total observations were discarded. An even sampling depth was used to assess alpha diversity and beta diversity measures. Beta diversity was assessed using weighted and unweighted UniFrac distance measures [77]. A rooted phylogenetic tree and rarefied feature tables were used to meet the assumptions of UniFrac. Ordination was performed using principal coordinate analysis (PCoA), and the statistical significance of group differences was assessed using permutational multivariate analysis of variance (PERMANOVA).

#### 2.6.7. Statistical Analysis

All animal data were summarized through descriptive statistics or frequency counts at each time interval with mean and standard deviation and/or incident counts (categorical variables) calculated for each endpoint. Data were analyzed using SAS Release 9.4 (SAS Institute, Cary, NC, USA). Body temperature, respiration rate, heart rate, body weight, hematology, coagulation, clinical chemistry, and urinalysis were analyzed by Repeated Measures Analysis of Covariance (RMANCOVA) with the fixed effects treatment (TRT), time (TIME), and sex (SEX); the 2-way interactions TRT•SEX, TRT•TIME, and TIME•SEX; and the 3-way interaction TRT•TIME•SEX and a covariate [78]. BCS, food consumption, and neurological examinations were analyzed by Repeated Measures Analysis of Variance (RMANOVA) with TRT, TIME, and SEX; the 2-way interactions TRT•TIME, TRT•SEX, and SEX•TIME; and the 3-way interaction (TRT•TIME•SEX)—all as fixed effects. Digestibility endpoints were analyzed by Analysis of Variance (ANOVA) using a model that included terms for TRT, SEX, and the TRT•SEX interaction. As the only endpoints that fell in this category were ratios, a generalized linear model was used. Differences in all tests were considered significant when *p* < 0.10. Phyla and genus relative abundance of microbiome data were analyzed using the mixed-model procedure of SAS (SAS Institute, Inc., Cary, NC, USA). The fixed effects of treatment (TRT), day, and their interaction (TRT × Day) were tested. Animals were considered a random effect. Least squares means were calculated for each variable across time points, with post hoc pairwise comparisons adjusted using Tukey’s method. Statistical significance was determined at a threshold of *p* < 0.05.

## 3. Results

### 3.1. Proximate Analysis and Feed Quality

The nutrient concentrations of each diet were similar in composition with a lower ash and protein content in the FK0 diet compared with the test diets. All other measures, including gross energy, fats, and fiber, were similar across all feeds. Minor differences in proximate components between samples analyzed prior to start of study (Table 2), twenty months after feed production at the end of the study (Table 3), and data used to calculate digestibility data (Table 4) reflect these timings as well as interlaboratory variation as a result of using different analytical labs (Midwest Labs for proximate analysis and University of Chicago for the digestibility study). All diets had complete AAFCO nutrient profile analyses performed and all diets met the AAFCO recommendations for adult dog maintenance [65], see Table 3. Additionally, the testing of feed samples for proximates, minerals, fatty acids, amino acids, and the vitamins B2 and B5, 20 months after production, confirmed that all diets still met AAFCO guidelines (Table 3). Hexanal levels were below the level of quantification (LOQ) whilst peroxide values were 14.5, 17.8, 19.1, and 17.9 mEq/kg fat for FK0, 4, 6, and 8, respectively, 20 months after production. Values for heavy metals (arsenic, cadmium, lead, and mercury) and aromatic hydrocarbons in the cultured protein were also <LOQ.

### 3.2. Food Consumption and Body Weight

Whilst the food offered was adjusted to maintain body weight and BCS through the study, food consumption was unaffected by treatment group. A statistically significant F test for the TRT•TIME interaction was found (*p* = 0.0436) due to an increase in daily consumption by week through the study. A concomitant increase in body weight was noted as a result (Table 5). However, this increase was within the ±10% difference in body weight planned as part of the study design.

Whilst FCR increased with increasing FK inclusion during the safety phase of the study, this trend was not reflected during the recovery phase, with the lowest FCR noted in the FK8 group. This pattern was also noted in FCR values for the whole study with the lowest FCR values in the FK0 group, increasing with increased inclusion levels. The weight gain rate showed a reduction with increasing FK inclusion during the safety phase as well as over the whole study, but it showed an increase in weight gain with increasing FK inclusion during the recovery phase.

BCSs were maintained between 2.0 and 3.5 for all groups with no statistically significant differences in BCS between groups or in individuals throughout the study.

### 3.3. Clinical Observations and Treatments

A small number of clinical observations was noted infrequently in both control and treatment groups, which was not considered to be related to the diet and constituted common findings for laboratory dogs of this age and breed. No statistical differences in body temperature, heart rate, respiration rate, or behavior between controls and treatment groups were noted during the course of the study. Additionally, no differences were noted in the ophthalmology or neurology observations, although a few findings were noted. A number of observations were made during the recovery phase including one FK0 female with absent righting reactions (a failure to regain the upright position when placed on the side), with one female and one male in the FK4 group and one male in the FK8 group showing decreased menace reflex (no blink response when the eye is approached with a hand), one female in the FK4 group with a decreased flexor reflex (the reduced withdrawal of a limb away from a stimulus), and one female in the FK0 group had an absent perineal reflex (a lack of anal sphincter contraction and tail flexion). These findings were considered incidental.

Veterinary treatments included the administration of 50–75 mg of carprofen for up to 3 days for two dogs, respectively, in each of the FK0, FK4, FK6, and FK8 treatment groups. One female in the FK4 group was noted with a small ranula under the tongue prior to the start of the study. On Day 92, this was excised under anesthesia with the dog being given 0.3 mL buprenorphine (0.3 mg/mL) for two days post surgery and amoxicillin trihydrate and clavulanate potassium tablets daily for 7 days post surgery. This female recovered uneventfully. One female dog in the FK8 treatment group was given supplemental feed in the form of canned food starting at Day 29 for one week at the request of the veterinarian due to inappetence. As this was one of the exclusion criteria for the study, she was not subject to further analysis but was kept in the study to minimize any welfare issues for her pair-housed partner.

Finally, all dogs were treated for a suspected nematode infection with 5 mg/kg pyrantel on Days 72 and 93.

### 3.4. Hematology and Coagulation

Blood parameters in this dog population were highly variable, leading to many differences in these measures over time and among diets (Table 6). No significant treatment or treatment interaction F tests were noted for red blood cell count, mean corpuscular hemoglobin concentration, or mean platelet volume. A statistically significant three-way interaction F test was noted for mean corpuscular volume (*p* = 0.0116), mean corpuscular hemoglobin (*p* = 0.0359), red blood cell distribution width (*p* = 0.0768), and platelet count (*p* = 0.0033). Significant F tests for the time by treatment interaction were found for hemoglobin (*p* = 0.0548) and hematocrit (*p* = 0.0563), whilst the reticulocyte count had a statistically significant main effect upon treatment F test (*p* = 0.0336). However, all of these measures remained within normal limits and followed a similar pattern, irrespective of inclusion level. In particular, changes noted in the test groups followed the same pattern as the control group (Table 6). Full data for males, females, and males and females combined are available in Supplementary Table S1.

For white blood cells, no statistically significant treatment or treatment interaction F tests were noted for lymphocyte counts, basophil counts, or large unstained cells. Significant F tests for the time by treatment interaction were found for white blood cells (*p* = 0.0004), neutrophil count (*p* = 0.0005), monocyte count (*p* = 0.0155), and eosinophil count (*p* = 0.0006). Values for white blood cell count, neutrophil count, and monocyte count showed a similar pattern, irrespective of dose, with an increase up to day 85 in all groups, a decline up to day 220, and a subsequent increase on day 232. Values for white blood cell count, neutrophil count, and monocyte count were above normal limits on day 85 for FK6 (white blood cell count: *p* = 0.0008; neutrophil count: *p* = 0.0007; monocyte count: *p* = 0.0005) and FK8 (white blood cell count: *p* = 0.0003; neutrophil count: *p* = 0.0002; monocyte count: *p* = 0.0033) but had returned to normal limits by the next sample point (day 169). Eosinophil counts remained within normal limits throughout the study for all groups.

Minor differences in activated partial thromboplastin time and prothrombin time were not statistically significant and, as they did not show a dose response, were considered normal (Table 7). An increase in fibrinogen in both males and females in FK4, FK6, and FK8 was noted up to day 85, after which it declined up to day 220. Whilst this increase showed a statistically significant (*p* = 0.0431) time-by-treatment interaction, it was still within normal limits for dogs at this stage and age, with the exception of the FK6 diet on day 85 (*p* = 0.0005), driven by a value of 316 mg/dL in the fibrinogen values for females (max value in reference range 305 mg/dL). A slight increase was noted in all groups at day 232 during the recovery phase. Full data for males, females, and males and females combined are available in Supplementary Table S2.

### 3.5. Clinical Chemistry

Ten of the chemistry endpoints had no statistically significant treatment or treatment interactions including gamma-glutamyltransferase, creatine kinase, creatinine, bicarbonate, bile acids, lactate dehydrogenase, cholesterol, triglycerides, sodium, and chloride (Table 8). In addition, four endpoints had statistically significant three-way interactions at the α = 0.05 level of significance and were thus excluded from pair-wise comparisons. These were AST (*p* = 0.0339), total bilirubin (*p* = 0.0417), blood urea nitrogen (*p* = 0.0275), and albumin (*p* = 0.0068). Significant time-by-treatment F tests were seen for ALT (*p* = 0.0100), alkaline phosphatase (*p* = 0.0093), glucose (*p* = 0.0188), and potassium (*p* = 0.0236). One analyte, phosphorus, showed a statistically significant F test for the sex-by-treatment interaction (*p* = 0.0914), with the difference being noted only in the females. Globulin had a statistically significant F test for the main effect of treatment (*p* = 0.0413). Changes in the various clinical chemistry endpoints showed a similar pattern, irrespective of dose. All those showing a statistically significant interaction maintained values within normal range with two exceptions—ALT with a value just above the normal range for FK8 on day 29, and phosphorus with values just below the normal range on days 169, 220, and 232 for FK0, FK6, and FK8. Full data for males, females, and males and females combined are available in Supplementary Table S3.

### 3.6. Urinalysis

Occasional differences in individual and mean urine volume, specific gravity, and pH that were not considered biologically meaningful were noted (Table 9). Urine volume had no statistically significant treatment-related effects or interactions. There was a statistically significant time-by-treatment F test for urine specific gravity (*p* = 0.0415), which was explained by males and females showing a change in specific gravity tested during the recovery phase (Day 204) in the FK6 group compared with the FK0 (*p* = 0.0028); values remained within normal limits. For urine pH, a significant treatment main effect was found (*p* = 0.0945). Pair-wise comparisons of treated group means relative to controls showed an increase in pH in males and females in FK4 (*p =* 0.0426) and in males and females in FK6 (*p* = 0.0331). There were some variations between dosed groups among physical (appearance), biochemical (occult blood, protein, etc.), and microscopic urinary components (red and/or white blood cells, bacteria, crystals, etc.); however, these findings were considered sporadic, consistent with biologic and procedure-related variation, and/or negligible in magnitude, and not related to treatment. Full data for males, females, and males and females combined are available in Supplementary Tables S4 and S5.

### 3.7. Apparent Total Tract Digestibility (ATTD)

Apparent total tract digestibility measurements of macronutrients and energy showed similar results irrespective of inclusion level with organic matter, protein, and energy having ATTD values above 80%, whilst ATTD values for fat exceeded 90% (Table 10). Although crude protein digestibility was not statistically significant between groups, there was a statistically significant F-test for the interaction between sex and treatment (*p* = 0.0376) which was explained by lower ATTD values for females in the FK4 group (ATTD = 79.51%, *p* = 0.0046) and the FK8 group (ATTD = 80.43%, *p* = 0.059) compared with the FK0 group (ATTD = 82.41%). Statistically significant differences were noted for organic matter (*p* = 0.0066), fat (*p* = 0.0067), and gross energy (*p* = 0.0012). For organic matter, fat, and gross energy, these were driven by marginally lower ATTD values in the FK4 and FK8 groups compared with the control group. Copper digestibility was lower in the treatment groups compared with the control group at around 20%, compared to 23.28% in FK0, but this was not statistically significant.

### 3.8. Fecal Microbiome

**Figure 1:**
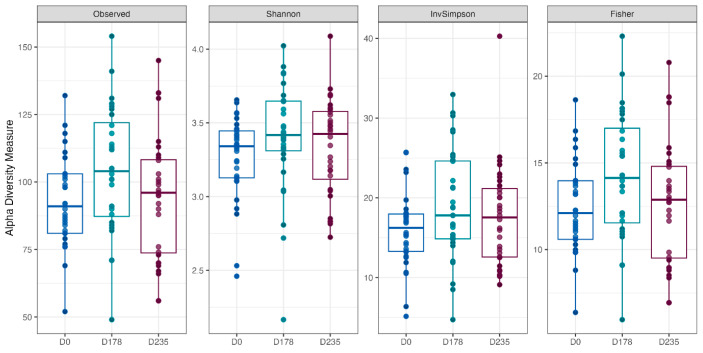
The alpha diversity of fecal samples from dogs across different time points (D0: baseline, D178: Day 178, D235: Day 235), illustrating variations in microbial richness and diversity over the study period.

**Figure 2:**
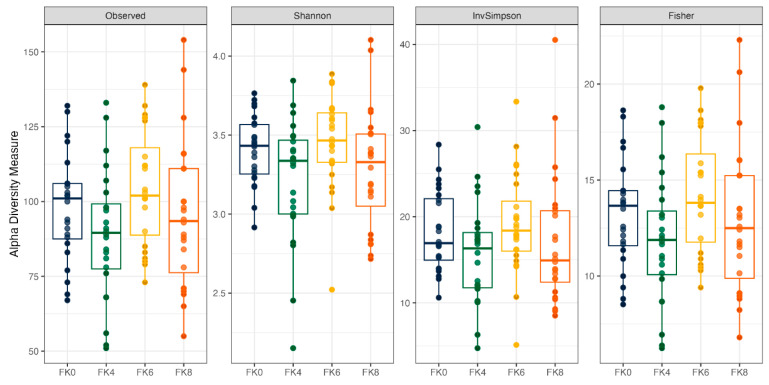
Alpha diversity of fecal samples from dogs across dietary treatments. Test diets included FeedKind^®^ (FK) at different inclusion levels: 0% (FK0), 4% (FK4), 6% (FK6), and 8% (FK8).

**Figure 3:**
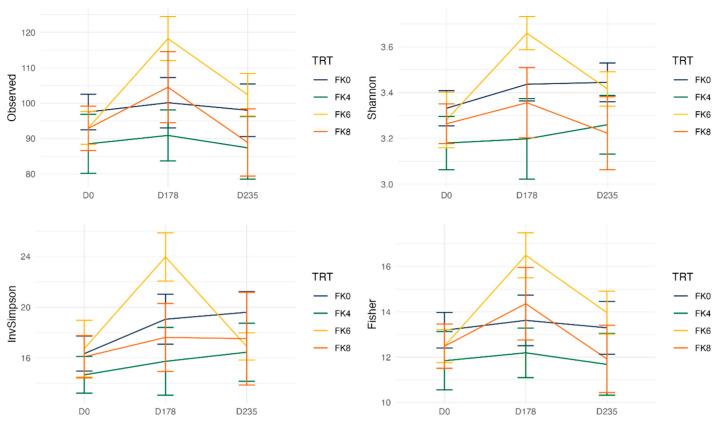
Alpha diversity of fecal samples from dogs, analyzed based on the interaction between dietary treatment and time point. Dietary treatments included FeedKind^®^ (FK) at 0% (FK0), 4% (FK4), 6% (FK6), and 8% (FK8), with samples collected at baseline (D0), Day 178 (D178), and Day 235 (D235).

**Figure 4:**
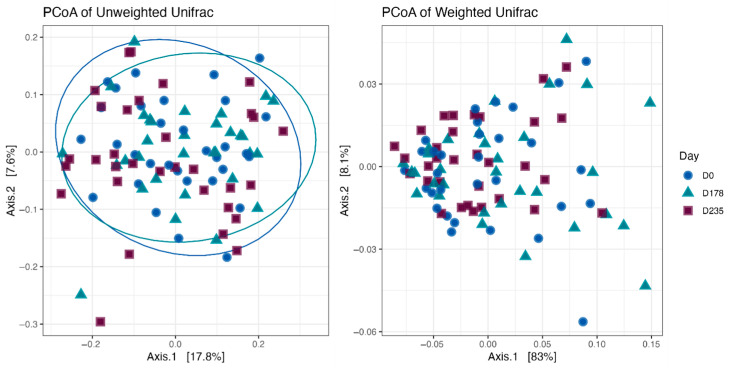
Beta diversity plots of fecal samples from dogs based on weighted and unweighted UniFrac distances at baseline (D0) and after 178 days (D178).

**Figure 5:**
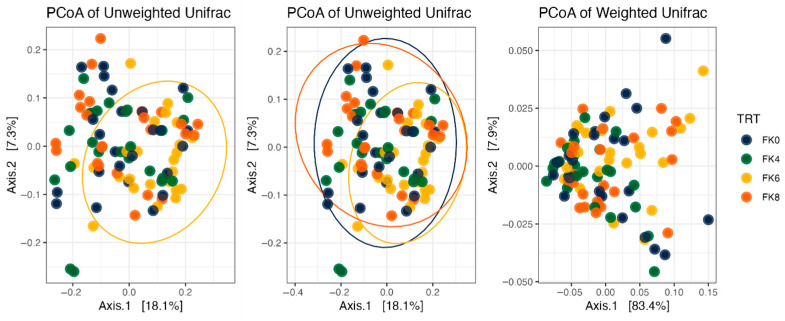
Beta diversity plots of fecal samples from dogs based on weighted and unweighted UniFrac distances. FK0: 0% FeedKind^®^ inclusion; FK4: 4% FeedKind^®^ inclusion; FK6: 6% FeedKind^®^ inclusion; FK8: 8% FeedKind^®^ inclusion.

**Figure 6:**
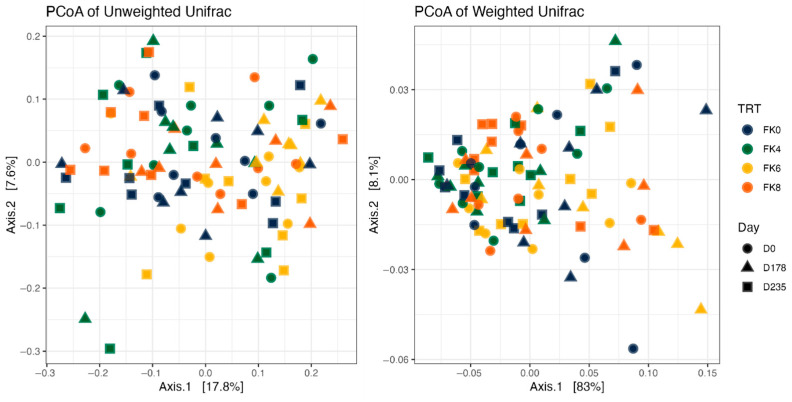
Beta diversity plots of fecal samples from dogs based on weighted and unweighted UniFrac distances. FK0: 0% FeedKind^®^ inclusion; FK4: 4% FeedKind^®^ inclusion; FK6: 6% FeedKind^®^ inclusion; FK8: 8% FeedKind^®^ inclusion. D0: baseline; D178: day 178.

Fecal alpha diversity was not significantly influenced by dietary treatment, time point, or their interaction (Figure 1, Figure 2 and Figure 3), although there was a general increase in all groups at the end of the safety phase, followed by a return to baseline at the end of the recovery phase. In contrast, beta diversity analysis using unweighted UniFrac distances showed distinct clustering patterns. Despite the lack of strong clustering, baseline (Day 0) and Day 178 (Figure 4) were different. FK0 was different to FK6 and FK8, but remained similar to FK4 (Figure 5). However, there was no significant effect of the interaction between dietary treatment and time point on beta diversity (Figure 6).

**Figure 7:**
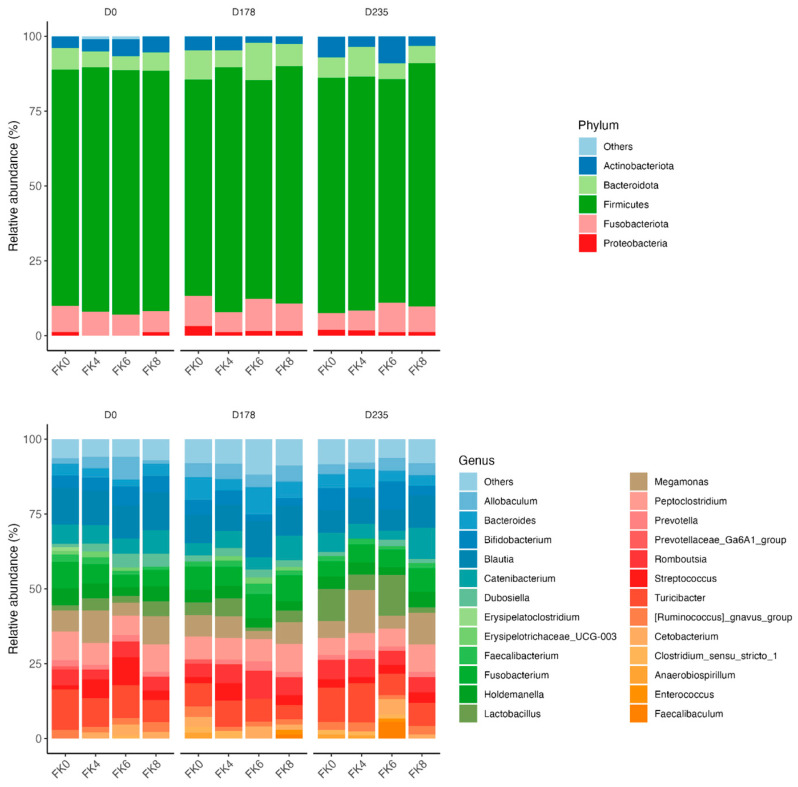
Relative abundance of fecal bacterial taxa in dogs across dietary treatments and time points. FK0: 0% FeedKind^®^ inclusion; FK4: 4% FeedKind^®^ inclusion; FK6: 6% FeedKind^®^ inclusion; FK8: 8% FeedKind^®^ inclusion. D0: baseline; D178: Day 178; D235: Day 235.

**Figure 8:**
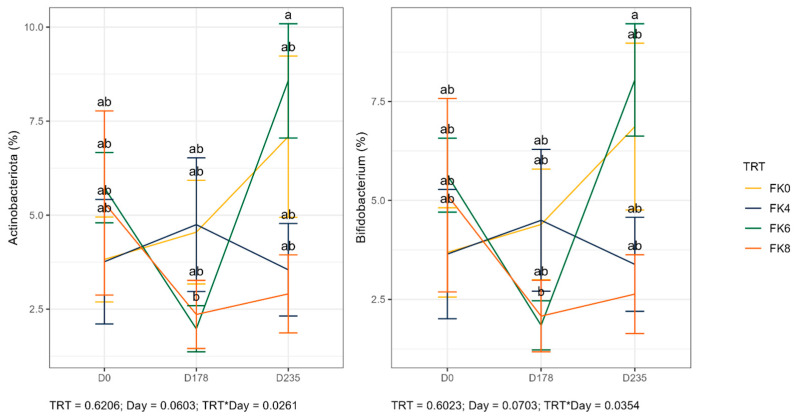
The relative abundance of the phylum Actinobacteria and its genus *Bifidobacterium* in the fecal samples of dogs across dietary treatments and time points. FK0: 0% FeedKind^®^ inclusion; FK4: 4% FeedKind^®^ inclusion; FK6: 6% FeedKind^®^ inclusion; FK8: 8% FeedKind^®^ inclusion. D0: baseline; D178: Day 178; D235: Day 235. Lower case letters represent significant differences (*p* < 0.05).

**Figure 9:**
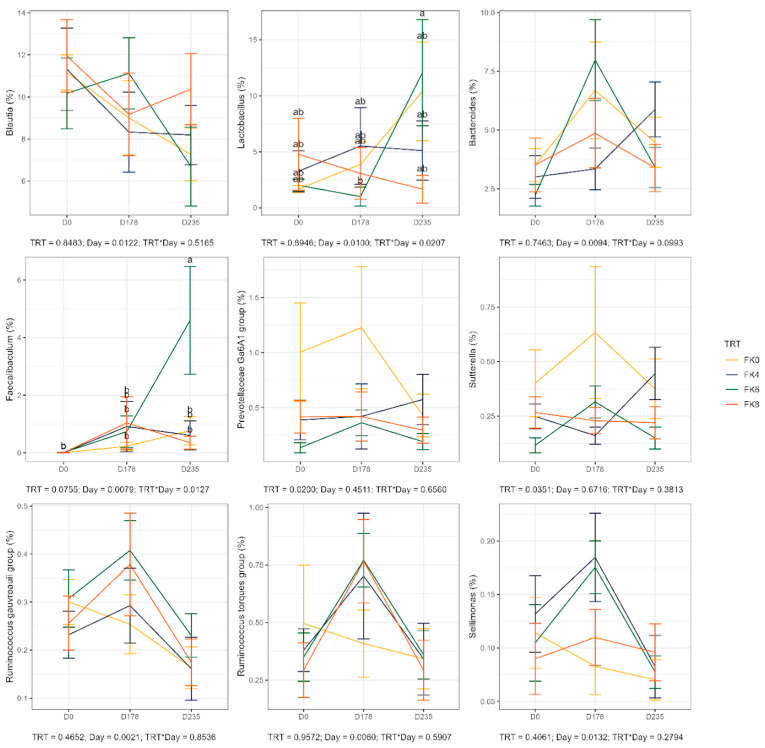
Relative abundance of specific bacterial taxa in fecal samples of dogs across dietary treatments and time points. FK0: 0% FeedKind^®^ inclusion; FK4: 4% FeedKind^®^ inclusion; FK6: 6% FeedKind^®^ inclusion; FK8: 8% FeedKind^®^ inclusion. D0: baseline; D178: Day 178; D235: Day 235. Lower case letters represent significant differences (*p* < 0.05).

The relative abundances (% of sequences) of predominant fecal bacterial phyla and genera of dogs in each dietary treatment across time points are presented in Figure 7. Fecal microbiota were dominated by Firmicutes in all groups, accounting for >75% of sequences on average across all time points and in all groups. Bacteroidota and Fusobacteria each accounted for ~10% of sequences. Euryarchaeota occurred in three dogs (at baseline day 0, in FK8 on Day 178, and FK6 on Day 235) at a relative abundance of less than 1%. The predominant genera included *Megamonas*, *Peptoclostridium*, *Turicibacter*, *Catenibacterium*, *Fusobacterium*, *Romboutsia*, and *Blautia* that occurred in almost all samples. *Lactobacillus*, *Bacteroides*, and *Allobaculum* occurred less frequently. The phylum Actinobacteria and its genus *Bifidobacterium* exhibited a significant interaction effect between dietary treatment and time point (TRT × Day). Specifically, FK6 had a lower abundance at Day 178 and a higher abundance at Day 235; however, it did not significantly differ from the other dietary treatments throughout the study (Figure 8). Additionally, Figure 9 highlights a significant dietary treatment effect for the *Prevotellaceae Ga6A1* group and *Sutterella*. FK0 displayed a higher relative abundance of the *Prevotellaceae Ga6A1* group compared to FK6, while *Sutterella* was more abundant in FK0 than in FK8 and FK6. An interaction effect was also observed in Figure 9, the relative abundance of *Lactobacillus* in FK6 was lower at Day 178 and higher at Day 235; however, it was not significantly different from the other dietary treatments throughout the study. Furthermore, *Faecalibaculum* abundance in FK6 at Day 235 was significantly higher than in all other dietary treatments. Full phyla and genera data for all dogs are available in Supplementary Tables S6 and S7.

## 4. Discussion

Previous studies in aquatic species, pigs, poultry, mink, and foxes have shown that whilst cultured protein derived from *Methylococcus capsulatus* is safe for use as an alternative protein source, the maximum inclusion level is species dependent with differential health and growth outcomes occurring with differing amounts of cultured protein in the diets. In the current study, all biomarkers for hematology, clinical chemistry, and urinalysis were within normal limits at the end of the safety phase for all groups with two exceptions, namely a decrease in phosphorus levels in the FK0, 6, and 8 groups, and a reduction in urine pH in all groups. As the values for hematology, clinical chemistry, and urinalysis are similar between all groups and within normal limits at the end of the study, they are considered to be normal for this group of dogs. As with other animal models, the current study has reaffirmed that cultured protein, in the form of FeedKind Pet, is safe for use in dog foods at up to 8% of the total diet.

Reduced feed intake in fish has been noted at high inclusion levels of cultured protein in the diet, limiting the maximum uptake of the protein in these animals [55]. In the current study, the four isonitrogenous, isolipidic, and isoenergetic diets containing increasing inclusions of FeedKind Pet were readily accepted by the dogs with no reductions in feed intake throughout the study. This ensured that the dogs received sufficient cultured protein to observe any potential effects over the six-month exposure period. Furthermore, the feed quality remained high as tested 20 months after production and whilst the peroxide values were elevated (14.5–19.1 mEq/kg fat), these occurred in all diets suggesting that this was not due to the inclusion of FeedKind Pet, rather that it being a function of the oxidation of other ingredients such as poultry fat. In a 20-day study by Babu et al. [79], dogs were fed increasing amounts of *M. capsulatus*, culminating in a diet containing up to 10% of the cultured protein for the final week of the study. No significant effects were noted in overall health, feed consumption, or fecal scores. Despite the short duration of that study, it is clear that the cultured protein is tolerated by dogs in the short and long term.

Although overall feed intake increased during the study, interpretation was complicated by adjustments in feed offered based on individual weight changes. Interestingly, whilst all groups gained weight over the study, the dogs fed kibble containing cultured protein showed a lower weight gain compared with the control group over the safety phase and an increase in weight gain once they were transferred to control diets during the recovery phase, partly attributed to higher FCR values in the cultured protein groups during the safety phase. These data are in contrast to those of Skrede & Ahlstrøm [52] who showed that up to 12% cultured protein in diets had no effect on body weight gain over the course of four months but did increase growth rates in blue foxes (*Vulpes lagopus*), which they attributed to improved FCR values. It is important to recognize that the current study was designed to assess safety over the study period, unlike typical nutrition studies where one of the main objectives is to maximize growth with the lowest FCR values. The reductions in weight gain rate in the FK groups were not reflected in lower body condition scores suggesting, at least based on weight and BCS, that the dogs were not unduly affected by the inclusion of cultured protein in the diets. Other than the significant statistical difference between start and end weights in all groups, there was no statistically significant difference in weights between groups over the study, confirming that assertion.

Digestibility values, particularly for dry matter, organic matter, and energy, were broadly comparable to those for other protein ingredient sources and are considered acceptable for dogs [80,81,82]. The digestibility coefficients of protein in the current study were comparable with those of soybeans [83]; lower than those of brewed lamb protein [84], duckweed [82], and plasma [80]; and higher than those of black soldier fly larvae [85] and shrimp hydrolysate [81]. Differences in total tract protein digestibility of cultured protein have been noted in pigs (85%) [60], foxes (84%) [53], and mink (78%) [51], but are broadly in line with the results from the current study. In a previous study, the digestibility of indispensable and non-indispensable amino acids in FK exceeded 85% and 80%, respectively [62]. Using AAFCO guidelines, DIASS-like scores were >100 for adult dogs and when combined with the current data for ATTD for macronutrients and energy, this demonstrates that cultured protein is a high-quality protein, able to meet the needs of a range of species, including dogs.

Concerns around the concentration and bioavailability of copper in dog foods have been raised as a result of apparent copper-associated hepatopathy (CAH) [86,87,88,89]. It is recognized that copper is an essential nutrient and is involved in a range of physiological processes as well as being immunomodulatory. Although there are minimum recommended levels of dietary copper of between 6 and 12 ppm in the total diet of dogs, there are no upper tolerable limits defined [65,90]. Some insect-based dog foods can contain up to 29.75 mg/kg of copper [91], similar to the maximum copper concentration in the current formulations of 29.5 mg/kg in the FK8 diet, and above the minimum recommended. FeedKind Pet contains around 83.67 mg/kg of copper, contributing approximately 6.69 mg/kg of copper in the FK8 formulation. With an ATTD for copper of approximately 20%, the maximum copper intake in the current study would therefore be around 6 mg/kg of feed, close to the minimum recommended values. Copper digestibility in other species fed diets containing 8–9% FK varies from 26.3% in Atlantic halibut [92], 65.8% in largemouth bass [93], and 40.9% in cats [94], demonstrating that copper digestibility is likely species dependent, requiring additional studies to determine values across the range of animal models. Despite levels of dietary copper in the current study of between 25 and 32 mg/kg of DM, this did not appear to elicit a host response with values for AST, alkaline phosphatase, and gamma-glutamyl transferase remaining within normal limits with no marked differences in values between the different formulations, demonstrating a lack of liver effect normally associated with CAH in the short term [86,87,88]. A marginal increase above normal baseline in ALT values in the FK8 group on Day 29 was considered transient as values returned to baseline by the following sample point and it is not likely that this was related to copper levels.

Measuring hematological parameters provides useful insights into potential effects on immune responses in animals exposed to potential toxicants, including those in new and novel food sources. In the current study, red blood cell parameters followed similar patterns across all groups, and although there was a statistically significant effect noted for hemoglobin, hematocrit, and reticulocyte counts, these remained within normal limits throughout the study. Interestingly, for all three measures, there was a reduction below baseline in all four groups by Day 85, followed by an increase above baseline by Day 169 during the safety phase. In the recovery phase, these values remained below baseline levels initially, then increased marginally by the end of the study. The presence or absence of cultured protein is unlikely to account for these changes as there was no marked difference in dog responses, even at different inclusion levels. However, it is interesting to speculate on the impact of the nematode infection in these dogs, treated with pyrantel on Days 72 and 93, either side of the blood samples taken on Day 85. Nematode infections can lead to reductions in hematocrit and hemoglobin levels [95] and, whilst speculative, may provide some explanation for the changes noted, particularly as these values showed the same pattern across all treatment groups. As all red blood cell measures fall within the normal ranges for beagles under laboratory conditions, they are not considered further.

In general, white blood cell parameters were within the normal range and no effect of treatment was observed. However, there was an increase in white blood cell counts, driven by increases in neutrophil, monocyte, and eosinophil counts, that showed a similar, but dose-dependent pattern, increasing up to Day 85 in all groups, declining up to Day 220, and subsequently increasing by Day 232. These measurements remained within normal limits with the exception of the FK6 and FK8 groups where white blood cell, neutrophil, and monocyte counts were above normal limits on Day 85. In particular, these transient shifts to higher white blood cell counts were driven by increases in neutrophil counts. A similar pattern was observed for fibrinogen, although values, with the exception of FK6 on Day 85, were within normal limits throughout the study. Increases in neutrophil counts and fibrinogen are indicative of an adaptive pro-inflammatory response. Dose-dependent inflammatory responses have been noted in fish fed on diets containing cultured protein [56,96,97,98,99,100], often providing useful protection against soybean meal-induced enteritis [56,96,97,101,102], as well as improving survival following bacterial infections [37]. Dose-dependent responses in an in vitro human whole-blood model, support the view that an inflammatory response is a normal reaction to cultured protein [103]. Increases in cultured protein-specific immunoglobulins, including IgA, IgG1, IgG2A, and total immunoglobulins in the blood were noted in mice fed cultured protein for eight weeks, and whilst the duration of the study was shorter than the current study, there was evidence of a decline in IgA titers in saliva towards the end of exposure [101]. It has been promulgated that the main driver for the inflammatory responses to cultured protein is either through exposure to dietary copper or lipopolysaccharide (LPS) in the bacteria [99,101]. Equally, exposure to novel antigens, such as those in cultured protein may elicit an immune response. LPS and other bacterial components are known to stimulate immune responses by interacting with innate immune pathways, leading to both pro-inflammatory and anti-inflammatory cytokine production. While Th-1-type cytokines can drive inflammatory responses and potential tissue damage if excessive, Th-2-type cytokines are generally associated with anti-inflammatory effects; both responses have been noted in mice and rats fed cultured protein [101,104]. Irrespective of the underlying mechanism, it is clear that the effect is transient and that dogs can tolerate cultured protein in their diets without the long-term activation of the immune system.

Endpoints for clinical chemistry were generally within normal limits and showed similar patterns, irrespective of inclusion level. Alanine aminotransferase (ALT) is a liver-specific enzyme in dogs, and it can be a useful indicator of liver damage, including inflammation [105,106]. ALT levels in the sera were just above the normal range for FK8 on Day 29, before returning to baseline levels by Day 85 when the values were similar to dogs fed test and control diets. The decrease in ALT levels in the current study following the initial increase suggests that any effect is transient and reversible. An increase in AST was also noted in the FK8 group, although this was within normal range. This initial doubling of ALT levels in the current study is certainly below those levels in dogs exhibiting severe hepatopathy [107] and has been noted in beagles fed standard diets [108]. The pattern of an initial ALT increase in the first six weeks followed by a subsequent decrease back to baseline levels in the Oba et al. [108] study was similar to the current study. In common with the current study, the dogs remained healthy.

Concerns surrounding the effect of feed-derived purines on animal health have been known for over a century [109,110], with particular issues noted in, e.g., dalmatians. Single-cell proteins contain variable amounts of RNA and DNA that, when digested, form purines or pyrimidines. It is the presence of purines that is of greater concern, given the link between purine levels and the production of urinary bladder stones or uroliths [111,112,113]. Degradation products from purine nucleotide metabolism include uric acid, responsible for the production of purine uroliths, and allantoin, a breakdown product of uric acid [113,114]. Allantoin accounted for around 97% of the excreted purine metabolites in mink fed diets containing 8% cultured protein [49]. Urine output and allantoin concentration were reduced, and uric acid excretion was increased in these mink compared with the control groups. In contrast, Hellwing et al. [50] showed that increasing levels of cultured protein in pigs and mink increased allantoin concentration in the urine of both species, but had no effect on uric acid excretion in mink. Reductions in urine output led the authors to conclude that renal function was not impaired and that cultured protein did not exert any urogenic effects in these animals. Dietary purines are utilized by the body to synthesize RNA and DNA, and can be excreted via feces as well as the urine [50]. Therefore, variations in urinary allantoin concentrations, whilst likely to be directly correlated with dietary purine levels, may be affected by the metabolic route used by the animal. FeedKind Pet has a purine (adenine and guanine) content of 2500 mg/100 g of dried product, broadly comparable to pork liver and baker’s yeast [114], contributing around 100 to 200 mg purines per 100 g in the FK4 and FK8 diets, respectively. Whilst purine metabolites were not directly measured in the urine of the dogs in the current study, the urinalysis data showed no adverse effects, including the presence of urinary crystals or uroliths, at any inclusion level. The role of the gut microbiome in purine metabolism in dogs has not been explored thus far but holds the promise of further elucidating how purines are used by animals [115]. The high relative abundance of Firmicutes may be responsible for the lack of effect by purines in the current study due to their role in purine metabolism [116]. Unlike dalmatians, which appear to lack the microbial activity required to degrade dietary purines [117], the absence of evidence of purine urolithiasis in beagles could suggest that they are able to partially or wholly metabolize dietary purines, most likely to allantoin. This hypothesis should be explored through further experimental studies.

The importance of the gastrointestinal microbiome in influencing gut health, immunity, growth, and metabolism is well recognized [118,119,120,121,122], and is reflected in efforts by the wider feed industry to assess the influence of diets on the microbiome. In the current study, the baseline fecal microbiome was determined at the start, and the influence of cultured protein on the microbiome functionality and plasticity was assessed by feeding the dogs diets containing cultured protein for six months followed by a two-month washout phase where they were fed a control diet. Although alpha diversity was not statistically different over the course of the study, there was an increase in these measures, particularly in the FK6 group after six months of feeding cultured protein, followed by a return to baseline once the dogs were fed control diets for two months. Similarly, alpha diversity increased in spotted seabass fed diets containing up to 8% FK when compared to diets containing soybean meal, responsible for soybean meal-induced enteritis in the same study [99]. However, the influence of diet manipulation on alpha diversity in dogs varies according to formulation. Whilst no differences in alpha diversity were noted in dogs fed hydrolyzed feather meal [123], there was an increase in alpha diversity following the addition of cereal fiber in adult dogs [124]. The addition of water to dry foods has been shown to increase alpha diversity, suggesting that these measures can be strongly influenced by a range of factors [125].

Variations in the fecal microbiome with time and treatment were noted. In general, increases in the relative abundance of a number of fecal bacteria were noted during the safety phase, although these were generally not statistically significant. Exceptions included an increase in *Prevotellaceae Ga6A1* and *Sutterella* in the FK0 groups and increases in *Ruminococcus torques* and *Sellimonas* in the treatment groups at the end of the safety phase. In agreement with the current study, reductions in the prevalence of Actinobacteriota and *Bifidobacterium*, and increases in Bacteroidota, *Bacteroides* and *Fusobacterium*, were noted by Babu et al. [79]. Despite the short duration and the gradual increase in dietary cultured protein content in that study, it is remarkable that these similarities exist, occur over the short and long term, and that *M. capsulatus* shows consistency in its effects on the fecal microbiome of dogs. One of the most notable differences between the groups in the current study was the increased weight gain rate in the control group compared with the FK groups, although no effect on health was seen. Increases in the relative abundance of *Prevotellaceae Ga6A1* and *Sutterella*, as seen in the FK0 group, are associated with obesity and weight gain [126]. Alongside the lower relative abundance of *Turicibacter* and increases in *R. torques* abundance in the FK groups, both of which are associated with controlled weight loss in obese dogs [108], provides tangible evidence for the role of the microbiome in controlling weight gain in these dogs. In the current study, however, feed was restricted and so this mechanism does not explain the reduced abundance of *Turicibacter* in the current study. *Turicibacter* produces short-chain fatty acids (SCFAs) in the intestine and, whilst reductions in their relative abundance in the FK groups may be a cause for concern, this is offset by increases in *Sellimonas* abundance which also produces SCFAs. Additionally, the increased abundance of *Ruminococcus torques* and *Sellimonas*, as noted on the FK groups, are useful markers of improved gut health.

## 5. Conclusions

The cultured protein, *Methylococcus capsulatus*, has been tested in adult dogs with no impact on health and growth measures at the end of the six-month exposure. The digestibility of FK was in line with previous studies and was comparable to control diets. Minor increases in white blood cell count in the early part of the exposure period were transient and likely reflect a normal response by dogs. Changes in the microbiome were noted and may have aided in weight maintenance and overall health outcomes. FeedKind Pet can therefore be safely included in adult dog foods at up to 8% of the diet. Further work on understanding its effects in younger dogs, and in cats, on elucidating the underlying mechanisms of cultured protein on physiological processes, and on the effect of increased doses on health outcomes should be conducted.

## Figures and Tables

**Table 1:** Proximate composition of FeedKind Pet^®^ cultured protein meal (% DM).

Crude Protein	74.68
Ash	7.17
Fat	8.48
Crude Fiber	0.03
Alanine	4.27
Arginine	3.89
Aspartic acid	5.21
Glutamic acid	6.51
Glycine	3.06
Histidine	1.34
Isoleucine	2.65
Leucine	4.61
Lysine	3.41
Phenylalanine	2.49
Proline	2.49
Serine	2.07
Threonine	2.62
Tyrosine	1.60
Valine	3.46
Tryptophan	0.77
Methionine	1.61
Cystine	0.37
Copper (mg/kg)	83.67

**Table 2:** Ingredient formulation of experimental diets as % of dry matter (DM).

	Diet			
Ingredient Name	FK0	FK4	FK6	FK8
FeedKind	0.000	4.000	6.000	8.000
Ground Corn	24.749	28.734	29.509	29.465
Soybean Meal	15.750	9.354	10.113	7.052
Chicken Meal	9.354	9.448	10.214	10.924
Corn Gluten Meal	9.354	9.262	6.000	5.000
Chicken Fat	7.671	7.247	7.091	7.052
Wheat Midds	7.671	7.247	7.091	7.052
Ground Wheat	7.671	7.247	6.324	7.052
Dicalcium Phosphate	3.777	3.602	3.436	3.524
Beet Pulp	3.200	2.974	3.465	4.000
Liquid Natural Flavor	3.000	3.000	3.000	3.000
Dry Natural Flavor	2.000	2.000	2.000	2.000
Blood Meal	1.000	1.000	1.000	1.000
Wheat Germ Meal	1.000	1.500	1.000	1.500
Fish Oil	0.652	0.649	0.635	0.482
Brewer’s Dried Yeast	0.635	0.397	0.200	0.200
Salt	0.610	0.572	0.538	0.515
Dried Whey	0.500	0.200	0.500	0.500
L-Threonine	0.301	0.322	0.311	0.323
Choline Chloride 60%	0.228	0.216	0.202	0.153
Potassium Chloride	0.222	0.371	0.716	0.552
Calcium Carbonate	0.200	0.200	0.200	0.200
Vitamins Premix	0.189	0.189	0.189	0.189
Minerals Premix	0.155	0.158	0.156	0.155
Mixed Tocopherols	0.096	0.096	0.096	0.097
BHA + BHT	0.015	0.015	0.015	0.015
Total value	100.000	100.000	100.000	100.000
Protein	28.35	28.91	29.39	30.40
Crude Fat	13.09	13.85	13.00	12.40
Crude Fiber	2.90	3.11	2.9	2.78
Ash	7.14	7.57	8.29	8.08

**Table 3:** Proximate analysis of experimental diets, 20 months after production (as-is values).

Feed Component	Units	FK0	FK4	FK6	FK8	Minimum Requirements ^1^
Moisture	%	7.28	7.50	8.21	8.24	-
Dry Matter	%	92.72	92.50	91.79	91.76	-
Crude Protein	%	25.80	27.40	28.30	27.90	22.50
Fat (acid hydrolysis)	%	13.90	11.00	11.60	10.40	8.50
Crude Fiber	%	4.02	4.77	3.42	2.98	-
Ash	%	6.32	6.88	7.41	7.34	-
Total Sulfur	%	0.33	0.36	0.37	0.37	-
Phosphorus (total)	%	1.20	1.31	1.37	1.38	1.00
Potassium (total)	%	0.95	0.99	1.13	1.02	0.60
Magnesium (total)	%	0.16	0.17	0.19	0.20	0.06
Calcium (total)	%	1.16	1.38	1.45	1.36	1.20
Sodium (total)	%	0.34	0.40	0.40	0.38	0.30
Iron (total)	ppm	324	384	356	356	88
Manganese (total)	ppm	53.00	56.00	55.70	63.80	7.20
Copper (total)	ppm	24.40	28.00	27.10	30.80	12.40
Zinc (total)	ppm	149	148	130	157	100
Aspartic acid	%	2.44	2.57	2.40	2.26	-
Threonine	%	1.11	1.19	1.18	1.19	1.04
Serine	%	1.23	1.30	1.20	1.18	-
Glutamic acid	%	4.90	4.86	4.62	4.20	-
Proline	%	1.81	1.76	1.75	1.43	-
Glycine	%	1.92	1.89	1.79	1.64	-
Alanine	%	1.88	1.98	1.89	1.84	-
Cystine	%	0.34	0.32	0.42	0.31	-
Valine	%	1.00	1.01	1.04	0.86	0.68
Methionine	%	0.43	0.45	0.50	0.50	0.35
Isoleucine	%	0.74	0.76	0.77	0.65	0.71
Leucine	%	1.70	2.16	2.07	2.20	1.29
Tyrosine	%	0.82	0.89	0.86	0.77	-
Phenylalanine	%	1.06	1.10	1.09	1.11	0.83
Lysine (total)	%	1.09	1.14	1.32	1.11	0.90
Histidine	%	0.59	0.62	0.61	0.56	0.44
Arginine	%	1.00	0.94	1.00	1.42	1.00
Tryptophan	%	0.23	0.26	0.27	0.28	0.20
Saturated fat (total)	g/100 g	4.28	3.41	3.63	3.28	-
Polyunsaturated fats (total)	g/100 g	3.25	2.62	2.75	2.45	-
Monounsaturated fats (total)	g/100 g	6.33	4.94	5.18	4.64	-
Trans fatty acids (total)	g/100 g	0.04	0.03	0.04	0.03	-
Omega 3 fatty acids (total)	g/100 g	0.22	0.19	0.22	0.18	-
Omega 6 fatty acids (total)	g/100 g	3.00	2.40	2.51	2.24	-
Omega 9 fatty acids (total)	g/100 g	5.39	4.19	4.38	3.92	-
Pantothenic acid (Vitamin B5)	mg/kg	39.8	45.9	57.6	61.4	12
Riboflavin (Vitamin B2)	mg/kg	5.26	5.94	6.77	6.72	5.2

^1^ Based on adult dog growth and reproduction minima according to AAFCO [65]. Maximum inclusion values for phosphorus and calcium are 1.60 and 2.50%, respectively, according to AAFCO.

**Table 4:** The analyzed chemical and energy composition of the diet fed to dogs; data calculated for the digestibility component.

Item	FK0	FK4	FK6	FK8
Dry matter, %	93.87	93.83	93.16	92.92
	Dry matter basis			
Organic matter	93.16	91.97	91.52	91.69
Ash	6.84	8.03	8.48	8.31
Crude protein	26.26	28.67	29.74	29.68
Acid-hydrolyzed fat	12.82	11.97	12.41	12.39
Total dietary fiber	16.04	16.62	15.98	16.27
Insoluble fiber	11.70	12.24	11.91	12.06
Soluble fiber	4.35	4.38	4.06	4.21
Gross energy, kcal/g	4.92	4.83	4.88	4.89

**Table 5:** Mean weight, weight gain rate, and feed conversion ratios (FCRs) of adult beagles fed diets containing 0% (FK), 4% (FK4), 6% (FK6), or 8% (FK8) FeedKind.

	FK0	FK4	FK6	FK8	*p*
Mean initial weight (kg) (day 0)	7.44 ± 1.372	7.70 ± 1.11	8.10 ± 1.24	8.24 ± 1.31	0.567
Mean final weight (kg) (day 175)	7.98 ± 1.61	8.10 ± 1.44	8.36 ± 1.49	8.27 ± 1.54	0.95
Mean final weight (kg) (day 239)	8.00 ± 1.62	8.23 ±1.49	8.60 ± 1.51	8.66 ± 1.45	0.789
Weight gain (%) (day 0 to 180)	7.23	4.85	3.24	0.35	-
FCR (day 0 to 180)	58.46	87.39	131.25	1238.66	-
Weight gain (%) (day 180 to 239)	0.16	0.77	2.08	4.30	-
FCR (day 181 to day 239)	776.81	163.33	63.37	32.23	-
Weight gain (%) (day 0 to 239)	7.56	6.47	6.17	5.03	-
FCR (day 0 to day 239)	73.13	85.96	91.08	113.22	-

**Table 6:** Hematology values (mean ± standard deviation) from dogs fed diets containing 0% (FK0, 4% (FK4), 6% (FK6), or 8% (FK8) FeedKind Pet for 6 months, followed by 2 months on diets containing 0% (FK0) FeedKind Pet. Data are shown for days 0, 169, and 232 to represent the samples taken at the end of each phase. The figures in bold represent values outside the normal range for these dogs. The values that were statistically significant at the level of α ≤ 0.05 when compared to the control values for the group are marked with *.

Day	Reference Range	FK0	FK4	FK6	FK8
Red Blood Cell Count (10^6^/µL)					
Day −7	5.64–7.98	6.831 ± 0.3171	6.828 ± 0.5255	6.929 ± 0.2923	7.051 ± 0.4330
Day 169		6.980 ± 0.5423	7.036 ± 0.4564	6.808 ± 0.4563	6.803 ± 0.3138
Day 232		6.679 ± 0.4660	6.734 ± 0.6398	6.904 ± 0.5460	6.936 ± 0.5195
Hemoglobin (g/dL)					
Day −7	13.2–18.3	15.90 ± 0.784	15.64 ± 1.089	16.25 ± 0.767	16.24 ± 1.021
Day 169		16.43 ± 1.236	16.46 ± 1.195	16.16 ± 1.072	15.66 ± 0.550
Day 232		15.84 ± 1.097	15.78 ± 1.655	16.38 ± 1.319	16.11 ± 1.122
Hemocrit (%)					
Day −7	39.8–55.5	46.18 ± 2.271	45.59 ± 2.966	47.05 ± 2.181	47.01 ± 2.718
Day 169		49.54 ± 3.398	50.40 ± 3.806	48.98 ± 3.184	47.70 ± 1.654
Day 232		47.33 ± 3.157	47.43 ± 4.756	49.24 ± 3.363	48.66 ± 3.123
Mean Corpuscular Volume (fL)					
Day −7	65.4–74.2	67.61 ± 2.325	66.80 ± 2.191	67.90 ± 1.058	66.66 ± 1.686
Day 169		71.09 ± 2.653	71.64 ± 2.571	71.96 ± 1.085	70.16 ± 1.969
Day 232		70.88 ± 2.575	70.40 ± 2.424	71.39 ± 1.118	70.21 ± 2.097
Mean Corpuscular Hemoglobin (pg)					
Day −7	21.6–24.6	23.31 ± 0.872	22.94 ± 0.905	23.42 ± 0.311	23.07 ± 0.687
Day 169		23.54 ± 0.865	23.40 ± 0.812	23.76 ± 0.437	23.01 ± 0.863
Day 232		23.71 ± 1.025	23.40 ± 0.741	23.71 ± 0.419	23.26 ± 0.812
Mean Corpuscular Hemoglobin Concentration (g/dL)					
Day −7	31.9–34.5	34.49 ± 0.203	34.34 ± 0.510	**34.54 ± 0.354**	**34.59 ± 0.302**
Day 169		33.14 ± 0.366	32.71 ± 0.409	33.01 ± 0.300	32.81 ± 0.481
Day 232		33.48 ± 0.341	33.26 ± 0.288	33.25 ± 0.563	33.11 ± 0.353
Red Blood Cell Distribution Width (%)					
Day −7	11.3–13.5	11.93 ± 0.369	12.06 ± 0.245	12.29 ± 0.327	11.96 ± 0.331
Day 169		12.43 ± 0.358	12.56 ± 0.346	12.90 ± 0.644	12.69 ± 0.422
Day 232		12.39 ± 0.519	12.11 ± 0.416	12.59 ± 0.506	12.31 ± 0.453
Platelet Count (10^3^/µL)					
Day −7	154–427	273.4 ± 31.75	269.9 ± 52.72	259.5 ± 50.61	276.4 ± 39.64
Day 169		293.6 ± 36.62	310.8 ± 50.32	330.4 ± 96.13	317.3 ± 43.33
Day 232		308.6 ± 51.90	294.3 ± 55.11	283.8 ± 62.97	327.3 ± 43.23
MPV (fL)					
Day −7	7.9–16.2	11.23 ± 1.557	10.78 ± 1.237	11.05 ± 1.232	10.29 ± 0.758
Day 169		11.98 ± 1.929	11.69 ± 2.209	12.34 ± 1.793	11.40 ± 1.349
Day 232		13.55 ± 1.535	13.13 ± 1.229	13.20 ± 1.605	12.87 ± 1.447
Reticulocytes (10^9^/L)					
Day −7	9.1–87.5	33.14 ± 5.901	32.43 ± 9.512	37.61 ± 9.101	38.57 ± 12.448
Day 169		31.35 ± 10.117	51.54 ± 19.799 *	57.74 ± 20.995 *	60.74 ± 38.094 *
Day 232		32.11 ± 7.260	35.90 ± 11.705	58.93 ± 26.505	59.31 ± 20.025
White Blood Cell Count (10^3^/µL)					
Day −7	5.59–13.33	8.503 ± 1.496	8.066 ± 1.113	8.544 ± 1.164	8.696 ± 1.260
Day 169		8.875 ± 1.098	10.829 ± 4.318	11.934 ± 4.146 *	12.973 ± 2.929 *
Day 232		9.414 ± 1.477	10.221 ± 1.656	10.080 ± 2.017	10.171 ± 0.756
Neutrophils (10^3^/µL)					
Day −7	3.02–9.19	4.844 ± 0.9667	4.683 ± 0.6899	4.810 ± 0.8069	5.286 ± 0.7566
Day 169		5.504 ± 0.9185	7.365 ± 3.8040	8.228 ± 3.4552 *	8.967 ± 1.9673 *
Day 232		5.840 ± 1.1491	6.505 ± 1.2514	6.096 ± 1.5059	6.566 ± 0.6471
Lymphocytes (10^3^/µL)					
Day −7	1.49–4.08	2.706 ± 0.6820	2.523 ± 0.4754	2.668 ± 0.2471	2.797 ± 0.6111
Day 169		2.453 ± 0.4245	2.423 ± 0.4086	2.635 ± 0.4800	2.774 ± 0.6780
Day 232		2.599 ± 0.4197	2.739 ± 0.3705	2.870 ± 0.4396	2.606 ± 0.3425
Monocytes (10^3^/µL)					
Day −7	0.2–0.87	0.318 ± 0.0785	0.369 ± 0.0815	0.420 ± 0.1042	0.460 ± 0.1393
Day 169		0.325 ± 0.0697	0.571 ± 0.4234	0.639 ± 0.3245	0.733 ± 0.2381 *
Day 232		0.430 ± 0.1836	0.449 ± 0.0620	0.520 ± 0.1343	0.487 ± 0.0711
Eosinophil (10^3^/µL)					
Day −7	0.08–0.74	0.515 ± 0.2244	0.403 ± 0.2149	0.545 ± 0.3349	0.310 ± 0.1363
Day 169		0.394 ± 0.1205	0.393 ± 0.2622	0.331 ± 0.1132	0.384 ± 0.4347
Day 232		0.464 ± 0.2391	0.445 ± 0.2478	0.498 ± 0.3409	0.419 ± 0.2168
Basophils (10^3^/µL)					
Day −7	0.02–0.15	0.064 ± 0.0239	0.043 ± 0.0219	0.049 ± 0.0125	0.056 ± 0.0299
Day 169		0.054 ± 0.0239	0.045 ± 0.0220	0.048 ± 0.0158	0.057 ± 0.0138
Day 232		0.054 ± 0.0130	0.053 ± 0.0271	0.059 ± 0.0181	0.064 ± 0.0382
Large Unstained Cells (10^3^/µL)					
Day −7	0.01–0.1	0.058 ± 0.0139	0.049 ± 0.0309	0.053 ± 0.0167	0.053 ± 0.0281
Day 169		0.035 ± 0.0160	0.033 ± 0.0219	0.051 ± 0.0336	0.059 ± 0.0363
Day 232		0.026 ± 0.0092	0.030 ± 0.0160	0.039 ± 0.0223	0.036 ± 0.0151

**Table 7:** Coagulation values (mean ± standard deviation) from dogs fed diets containing 0% (FK0, 4% (FK4), 6% (FK6), or 8% (FK8) FeedKind Pet for 6 months, followed by 2 months on diets containing 0% (FK0) FeedKind Pet. Data are shown for days 0, 169, and 232 to represent the samples taken at the end of each phase. No values were outside the normal range for these dogs at these time points.

Days	Reference Range	FK0	FK4	FK6	FK8
Activated Partial Thromboplastin Time (s)					
Day −7	9.8–13.8	12.49 ± 0.409	12.36 ± 0.717	12.68 ± 0.406	12.54 ± 0.556
Day 169		12.18 ± 0.459	12.05 ± 0.798	12.28 ± 0.471	12.21 ± 0.363
Day 232		12.19 ± 0.380	12.01 ± 0.596	12.40 ± 0.421	12.40 ± 0.370
Fibrinogen (mg/dL)					
Day −7	144–305	164.6 ± 14.09	185.4 ± 42.73	191.0 ± 26.15	184.4 ± 39.31
Day 169		188.0 ± 44.15	206.4 ± 55.32	249.0 ± 64.00	241.4 ± 54.52
Day 232		184.6 ± 27.76	226.8 ± 47.20	222.3 ± 34.82	221.4 ± 68.19
Prothrombin Time (s)					
Day −7	7.0–8.8	8.16 ± 0.262	8.00 ± 0.256	7.88 ± 0.175	8.29 ± 0.219
Day 169		7.98 ± 0.341	7.93 ± 0.191	7.75 ± 0.330	8.13 ± 0.287
Day 232		8.10 ± 0.302	7.83 ± 0.276	7.80 ± 0.193	8.21 ± 0.261

**Table 8:** Clinical chemistry values (mean ± standard deviation) from dogs fed diets containing 0% (FK0, 4% (FK4), 6% (FK6), or 8% (FK8) FeedKind Pet for 6 months, followed by 2 months on diets containing 0% (FK0) FeedKind Pet. Data are shown for days 0, 169, and 232 to represent the samples taken at the end of each phase. Figures in bold represent values outside the normal range for these dogs.

Days	Reference Range	FK0	FK4	FK6	FK8
Albumin/Globulin Ratio					
Day −7	1.1–1.9	1.61 ± 0.146	1.54 ± 0.233	1.73 ± 0.413	1.56 ± 0.113
Day 169		1.39 ± 0.146	1.33 ± 0.301	1.30 ± 0.169	1.17 ± 0.180
Day 232		1.29 ± 0.125	1.19 ± 0.189	1.24 ± 0.151	1.13 ± 0.138
Albumin (g/dL)					
Day −7	2.9–3.6	3.30 ± 0.251	3.34 ± 0.141	3.31 ± 0.181	3.36 ± 0.113
Day 169		3.25 ± 0.227	3.29 ± 0.230	3.23 ± 0.149	3.21 ± 0.195
Day 232		3.05 ± 0.169	3.04 ± 0.192	3.09 ± 0.155	3.07 ± 0.198
Alkaline Phosphatase (U/L)					
Day −7	22–126	55.1 ± 15.48	62.0 ± 22.13	56.3 ± 13.44	57.6 ± 12.12
Day 169		60.0 ± 26.34	59.6 ± 25.04	56.0 ± 12.02	66.7 ± 21.91
Day 232		53.4 ± 16.93	59.5 ± 26.06	54.3 ± 14.74	60.9 ± 17.85
Alanine Transferase (U/L)					
Day −7	19–59	31.8 ± 7.48	29.0 ± 2.62	30.9 ± 6.69	32.6 ± 3.60
Day 169		33 ± 6.82	33.0 ± 6.02	34.8 ± 6.71	39.3 ± 19.09
Day 232		30.3 ± 10.08	27.8 ± 4.68	29.0 ± 4.60	30.4 ± 7.48
Aspartate Aminotransferase (U/L)					
Day −7	20–47	30.0 ± 3.7	31.0 ± 5.10	33.1 ± 5.51	32.4 ± 6.11
Day 169		31.4 ± 7.42	35.1 ± 4.36	36.1 ± 11.58	37.1 ± 12.06
Day 232		29.6 ± 4.21	31.4 ± 2.26	35.1 ± 4.76	34.0 ± 6.81
Bile acids (umol/L)					
Day −7	No reference range	0.54 ± 0.346	0.63 ± 0.568	0.66 ± 0.424	1.56 ± 1.693
Day 169		0.83 ± 0.623	4.31 ± 8.017	3.05 ± 4.407	2.13 ± 2.336
Day 232		2.01 ± 1.132	4.15 ± 8.765	5.61 ± 9.888	1.16 ± 0.787
Calcium (mg/dL)					
Day −7	9.4–11.0	10.06 ± 0.267	10.05 ± 0.177	9.93 ± 0.219	10.14 ± 0.251
Day 169		9.94 ± 0.160	10.14 ± 0.389	9.99 ± 0.304	9.94 ± 0.282
Day 232		9.58 ± 0.183	9.74 ± 0.374	9.83 ± 0.271	9.71 ± 0.273
Cholesterol (mg/dL)					
Day −7	104–252	159.4 ± 44.78	163.9 ± 17.68	171.0 ± 46.50	154.7 ± 25.17
Day 169		163.5 ± 35.61	149.9 ± 28.01	158.8 ± 35.79	153.9 ± 27.72
Day 232		181.8 ± 47.98	161.1 ± 17.33	182.8 ± 46.60	168.6 ± 52.18
Creatine Kinase (U/L)					
Day −7	81–458	180.9 ± 48.92	162.0 ± 34.14	209.9 ± 63.31	201.6 ± 81.39
Day 169		181.0 ± 127.65	156.0 ± 57.03	182.6 ± 173.33	183.0 ± 93.04
Day 232		159.6 ± 45.06	163.9 ± 53.91	227.0 ± 103.84	207.1 ± 98.05
Chloride (mEq/L)					
Day−7	109–117	115.0 ± 1.20	115.0 ± 1.60	114.9 ± 1.46	115.3 ± 1.38
Day 169		114.5 ± 1.77	115.0 ± 1.31	114.1 ± 2.03	114.3 ± 1.98
Day 232		115.5 ± 1.60	116.4 ± 1.41	115.5 ± 1.85	116.1 ± 1.57
Bicarbonate (mEq/L)					
Day −7	No reference range	23.9 ± 3.91	22.4 ± 3.66	21.6 ± 2.20	22.0 ± 2.83
Day 169		22.0 ± 2.07	20.4 ± 1.77	21.6 ± 1.85	22.4 ± 4.28
Day 232		22.0 ± 1.07	21.6 ± 2.33	20.4 ± 2.13	20.6 ± 2.57
Creatinine (mg/dL)					
Day −7	0.4–0.8	0.58 ± 0.0171	0.63 ± 0.071	0.60 ± 0.076	0.63 ± 0.076
Day 169		0.61 ± 0.064	0.66 ± 0.130	0.61 ± 0.136	0.59 ± 0.069
Day 232		0.61 ± 0.064	0.66 ± 0.092	0.63 ± 0.089	0.61 ± 0.069
Gamma Glutamyl Transferase (U/L)					
Day −7	0.0–4.0	0.0 ± 0.0	0.0 ± 0.0	0.0 ± 0.0	0.0 ± 0.0
Day 169		0.0 ± 0.0	0.0 ± 0.0	0.0 ± 0.0	0.0 ± 0.0
Day 232		0.0 ± 0.0	0.0 ± 0.0	0.0 ± 0.0	0.0 ± 0.0
Globulin (g/dL)					
Day −7	1.7–2.9	2.06 ± 0.213	2.20 ± 0.245	2.00 ± 0.298	2.20 ± 0.191
Day 169		2.36 ± 0.262	2.56 ± 0.472	2.54 ± 0.288	2.77 ± 0.315
Day 232		2.40 ± 0.200	2.58 ± 0.358	2.51 ± 0.189	2.70 ± 0.231
Glucose (mg/dL)					
Day −7	67–101	88.1 ± 9.51	87.8 ± 5.55	87.6 ± 8.00	86.7 ± 6.60
Day 169		90.8 ± 8.41	83.0 ± 7.13	80.5 ± 6.35	80.0 ± 9.24
Day 232		89.3 ± 8.31	86.3 ± 7.54	83.3 ± 7.11	85.6 ± 5.77
Potassium (mEq/L)					
Day −7	4.0–4.9	4.34 ± 0.169	4.26 ± 0.185	4.30 ± 0.200	4.37 ± 0.256
Day 169		4.31 ± 0.210	4.36 ± 0.192	4.40 ± 0.227	4.30 ± 0.265
Day 232		4.29 ± 0.146	4.35 ± 0.298	4.53 ± 0.198	4.60 ± 0.283
Lactate Dehydrogenase (U/L)					
Day −7	40–303	171.5 ± 67.00	139.8 ± 77.57	220.5 ± 91.60	168.0 ± 100.07
Day 169		169.6 ± 152.13	134.5 ± 86.27	199.5 ± 268.72	155.7 ± 145.04
Day 232		141.0 ± 82.99	111.8 ± 51.84	218.5 ± 130.12	152.6 ± 60.87
Sodium (mEq/L)					
Day −7	144–150	147.3 ± 0.46	147.3 ± 1.04	146.4 ± 0.74	147.7 ± 0.95
Day 169		146.3 ± 1.49	146.0 ± 1.07	145.8 ± 2.31	146.0 ± 1.29
Day 232		146.8 ± 1.49	146.9 ± 1.13	146.4 ± 1.30	146.6 ± 0.49
Phosphorus (mg/dL)					
Day −7	3.2–5.4	4.01 ± 0.270	4.13 ± 0.337	3.80 ± 0.342	4.06 ± 0.310
Day 169		**3.01 ± 0.236**	3.34 ± 0.220	**3.14 ± 0.307**	**3.04 ± 0.565**
Day 232		**2.85 ± 0.283**	**2.90 ± 0.389**	**3.18 ± 0.474**	**2.96 ± 0.336**
Total Bilirubin (mg/dL)					
Day −7	0.0–0.1	0.016 ± 0.0119	0.014 ± 0.0106	0.011 ± 0.0173	0.017 ± 0.0150
Day 169		0.030 ± 0.0185	0.021 ± 0.0155	0.018 ± 0.0167	0.013 ± 0.0111
Day 232		0.029 ± 0.0196	0.018 ± 0.0231	0.015 ± 0.0141	0.017 ± 0.0150
Total Protein (g/dL)					
Day −7	4.8–6.2	5.36 ± 0.385	5.54 ± 0.239	5.31 ± 0.280	5.56 ± 0.257
Day 169		5.61 ± 0.432	5.85 ± 0.460	5.76 ± 0.226	5.99 ± 0.195
Day 232		5.45 ± 0.267	5.61 ± 0.376	5.60 ± 0.177	5.77 ± 0.304
Triglycerides (mg/dL)					
Day −7	19–65	26.9 ± 7.66	30.9 ± 5.49	34.6 ± 10.85	29.9 ± 8.05
Day 169		29.6 ± 6.35	34.3 ± 4.06	36.4 ± 7.37	36.4 ± 7.35
Day 232		30.3 ± 5.23	35.0 ± 6.82	38.8 ± 5.28	36.3 ± 10.44
Urea Nitrogen (mg/dL)					
Day −7	10.0–24.0	13.0 ± 1.93	13.4 ± 1.69	13.1 ± 1.36	13.3 ± 1.70
Day 169		12.0 ± 1.20	13.0 ± 2.93	13.6 ± 1.92	12.1 ± 1.35
Day 232		11.3 ± 1.04	11.6 ± 1.77	11.6 ± 1.06	11.6 ± 1.81

**Table 9:** Urinalysis values (mean ± standard deviation) from dogs fed diets containing 0% (FK0, 4% (FK4), 6% (FK6), or 8% (FK8) FeedKind Pet for 6 months, followed by 2 months on diets containing 0% (FK0) FeedKind Pet. Data are shown for days 0, 169, and 232 to represent the samples taken at the end of each phase. Figures in bold represent values outside the normal range for these dogs. Values that were statistically significant at the level of α ≤ 0.05 when compared to the control values for the group are marked with *.

Day	Reference Range	FK0	FK4	FK6	FK8
Volume (mL)					
Day −7	No reference range	93.98 ± 66.272	86.45 ± 55.112	81.26 ± 31.135	125.14 ± 123.567
Day 169		123.20 ± 101.446	99.53 ± 58.867	107.04 ± 64.784	166.40 ± 172.987
Day 232		91.85 ± 50.951	56.50 ± 48.977	89.23 ± 58.860	121.26 ± 78.126
Specific gravity					
Day −7	1.010–1.070	1.0480 ± 0.0201	1.0494 ± 0.0175	1.0489 ± 0.0102	1.0463 ± 0.0278
Day 169		1.0396 ± 0.0205	1.0370 ± 0.0125	1.0366 ± 0.0101	1.0329 ± 0.0209
Day 232		1.0399 ± 0.0144	1.0451 ± 0.0113	1.0361 ± 0.0165	1.0333 ± 0.0166
pH					
Day −7	5.50–9.00	**5.470 ± 0.1797**	**5.365 ± 0.1259**	**5.485 ± 0.4294**	5.601 ± 0.5632
Day 169		5.565 ± 0.3307	**5.328 ± 0.1681** *	**5.368 ± 0.1349** *	5.591 ± 0.4281
Day 232		**5.426 ± 0.2026**	**5.260 ± 0.2089**	**5.311 ± 0.3629**	**5.309 ± 0.1586**

**Table 10:** Apparent total tract macronutrient and energy digestibilities, food and energy intake, and fecal output of dogs. Where FK0, FK4, FK6, FK8 = FeedKind inclusion levels, 0–8%; ^1^ SEM = pooled standard error of the means, DM = dry matter, OM = organic matter. Superscript lower-case letters reflect statistically similar values.

Item	FK0	FK4	FK6	FK8	SEM ^1^	*p*-Value
Food intake						
g food/d, as-is	169.00	179.32	191.23	197.34	7.45	0.0581
g food/d, DM	158.63	168.26	178.15	183.37	6.96	0.0865
kcal/d, as-is	781.25	813.12	870.25	897.43	34.10	0.0941
Fecal output						
g/d, as-is	107.75	133.17	122.66	130.28	8.00	0.1302
g/d, DM	31.47 ^b^	36.82 ^ab^	35.40 ^ab^	39.21 ^a^	1.41	0.0060
Digestibility, %						
DM	80.11	78.07	76.35	78.56	1.95	0.5893
OM	82.92 ^a^	80.94 ^b^	83.21 ^a^	81.63 ^ab^	0.48	0.0066
Fat	91.92 ^a^	90.55 ^b^	91.93 ^a^	91.44 ^ab^	0.29	0.0067
Protein	81.09	80.23	81.68	80.25	0.53	0.1789
Energy	83.78 ^a^	81.83 ^b^	83.78 ^a^	82.35 ^ab^	0.38	0.0012
Copper	23.28	19.59	20.71	20.11	2.01	0.5879

## Data Availability

The original contributions presented in this study are included in the article/Supplementary Materials. Further inquiries can be directed to the corresponding author.

## Supplementary Materials

The following supporting information can be downloaded at: https://www.mdpi.com/article/10.3390/ani15131975/s1, Table S1: Hematology of dogs fed diets containing cultured protein (*Methylococcus capsulatus*); Table S2: Coagulation values of dogs fed diets containing cultured protein (*Methylococcus capsulatus*); Table S3: Clinical chemistry values of dogs fed diets containing cultured protein (*Methylococcus capsulatus*); Table S4: Urinalysis values of dogs fed diets containing cultured protein (*Methylococcus capsulatus*); Table S5: Urine microscopy results of dogs fed diets containing cultured protein (*Methylococcus capsulatus*); Table S6: Phyla-level fecal microbiome of dogs fed diets containing cultured protein (*Methylococcus capsulatus*); Table S7: Genera-level fecal microbiome of dogs fed diets containing cultured protein (*Methylococcus capsulatus*).

## Abbreviations

The following abbreviations are used in this manuscript:

AOAC	Association of Official Analytical Chemists
BHA + BHT	Butylated Hydroxyanisole and Butylated Hydroxytoluene
DIAAS-like	Digestible, indispensable amino acid score
EDTA	Ethylenediaminetetraacetic acid
fL	Femtoliters
g/dL	Grammes per deciliter
HCl	Hydrochloric acid
HPLC	High-performance liquid chromatography
ICP-MS	Inductively coupled plasma mass spectrometry
LC-PUFA	Long-chain polyunsaturated fatty acids
µL	Microliter
mEq/kg	Milliequivalents per kilogram
mEq/L	Milliequivalents per liter
mg/dL	Milligrams per liter
pg	Picograms
U/L	Units per liter
